# Supplementary material for: Oncogenic mutant RAS signaling activity is rescaled by the ERK/MAPK pathway
Source: Mol Syst Biol. 2020 Oct 19;16(10):e9518. doi: 10.15252/msb.20209518 (PMC7569415; doi:10.15252/msb.20209518)
Supplement: Supplementary file 1 — Appendix [file MSB-16-e9518-s001.pdf]

Appendix

Oncogenic mutant RAS activity is constrained by signal rescaling in the ERK/MAPK pathway

Taryn E. Gillies[1*], Michael Pargett[1*], Jillian M. Silva[2], Carolyn Teragawa[1], Frank McCormick[2,3], and John G. Albeck[1]

[1] Department of Molecular and Cellular Biology, University of California, Davis, CA

[2] UCSF Helen Diller Family Comprehensive Cancer Center, San Francisco, CA

[3]Frederick National Laboratory for Cancer Research, Frederick, MD, USA

[*]Equal contributions

Table of Contents

Appendix Methods 1

FRET reporter measurement and correction (EKAR) 1

Inferring ERK activity from EKAR 3

RAS-ERK internal factors model 6

Western blotting supplemental methods 6

Appendix Table S1: Literature-based values for RAS related biochemical parameters 7

Appendix Methods

FRET reporter measurement and correction (EKAR)

To quantify the measurements of EKAR, we employ a spectral model of light propagated through the microscopy system, including the live cell specimen. In our microscope, light is generated via LEDs and passed through spectral excitation filters to illuminate the specimen. The specimen then fluoresces and the resulting light is collected by the objective and passed through spectral emission filters before illuminating the camera. We therefore, define a spectral model including all elements that modify the spectrum: (1) light source, (2) excitation filters, (3) fluorophores in the specimen, (4) emission filters, and (5) camera sensor. For a given pixel, the model defines the pixel intensity as the product of: light source intensity $I_{LS}(\lambda)$, excitation filter transmissivity $F_{ex}(\lambda)$, specimen absorbance $A_S(\lambda)$, specimen concentration C_S, specimen quantum yield QY_S, specimen emission spectrum $\phi_S(\lambda)$, geometric fraction of light collected by the objective G_{geo}, emission filter transmissivity $F_{em}(\lambda)$, camera quantum efficiency $QE_{cam}(\lambda)$, loss to absorbance/scattering from all optics G_{opt}, and time of exposure t. All terms that do not have a uniform effect across the spectrum are shown as dependent on wavelength λ.

$$I(\lambda) = \underbrace{I_{LS}(\lambda)F_{ex}(\lambda)A_S(\lambda)}_{X}\, C_S\, \underbrace{QY_S\phi_S(\lambda)F_{em}(\lambda)QE_{cam}(\lambda)}_{M}\, G_{geo}G_{opt}t \qquad (1)$$

The linearity in absorption by the specimen implies the assumption that the concentration of specimen is low enough for the exponential in the Beer-Lambert law (transmissivity, $T(\lambda) = 10^{-A(\lambda)c}$) to be approximately linear. The model may be simplified slightly by lumping all uncertain gain terms (geometric collection G_{geo}, optical loss G_{opt}, light source average power, electronic gain in camera circuitry, any other inefficiencies, etc.) to a single term $G = G_{geo}G_{opt}G_{ls}G_{cam} \ldots$. All terms must be determined to fully calibrate the model and calculate the absolute concentration of fluorophore in the specimen.

Including the effect of a FRET interaction between two fluorophores in this model gives a general model, suitable for any spectral collection channels (Hoppe et al., 2002). This equation now includes the integration over the spectrum, yielding the measured pixel intensity. Though not explicitly shown in notation

from here on, all fluorophore-specific terms (X^D, M^A, etc.) are dependent on wavelength. Superscripts D and A refer to the Donor and Acceptor molecules, respectively (mTurquoise2, and YPet in the case of the EKAR3 sensor we use). Note that while the EKAR reporters are single molecules with two fluorophores, the concentration of the donor (C^D) and acceptor (C^A) can be considered separately, accounting for truncated proteins and bleached or denatured fluorophores, which may vary from cell to cell and across cultures.

$$I = Pt \int \overbrace{C^D X^D M^D (1 - }^{Donor\ Signal} \overbrace{Ef_A) + C^D X^D M^A Ef_A}^{FRET\ Effect} + \overbrace{C^A X^A M^A}^{Acceptor\ Signal} \quad d\lambda \tag{2}$$

Our measurement of FRET sensors involves recording two spectral channels, CFP and YFP; the donor-exciting YFP channel essentially accounts for the expression level of the sensor. By considering a ratio of these channels, gain terms that are common to both channels are eliminated, leaving the spectral terms, as well as exposure time and average light source power if these varied between channels. The ratio of CFP to YFP is then represented by the following.

$$\frac{I_{CFP}}{I_{YFP}} = \frac{P_{CFP} t_{CFP} \int \left(C^D X^D_{CFP} M^D_{CFP} (1 - Ef_A) + C^D X^D_{CFP} M^A_{CFP} Ef_A + C^A X^A_{CFP} M^A_{CFP}\right) d\lambda}{P_{YFP} t_{YFP} \int \left(C^D X^D_{YFP} M^D_{YFP} (1 - Ef_A) + C^D X^D_{YFP} M^A_{YFP} Ef_A + C^A X^A_{YFP} M^A_{YFP}\right) d\lambda} \tag{3}$$

Because the CFP and YFP filter sets we employ are spaced spectrally such that CFP and YFP are not cross-excited, $X^D_{YFP} = 0$, $M^A_{CFP} = 0$, simplifying the model.

$$\frac{I_{CFP}}{I_{YFP}} = \frac{(1 - Ef_A) C^D P_{CFP} t_{CFP} \int (X^D_{CFP} M^D_{CFP}) d\lambda}{C^A P_{YFP} t_{YFP} \int (X^A_{YFP} M^A_{YFP}) d\lambda} \tag{4}$$

Solving for the fraction associated yields a function of the CFP-to-YFP ratio and a correction term, which can be divided into two factors: R_C, the ratio of active Donors to Acceptors, which may vary from cell to cell due to bleaching and translation defects, and R_P, the ratio of imaging power per channel, which is a constant per experiment. The FRET efficiency for EKAR3 (E) is not known, but has a strictly linear effect, leaving relative fractions associated accurate but absolute values uncertain.

$$Ef_A = 1 - \frac{I_{CFP}}{I_{YFP}} \Bigg/ \left(\underbrace{\frac{C^D}{C^A}}_{R_C} \underbrace{\frac{P_{CFP} t_{CFP} \int (X^D_{CFP} M^D_{CFP}) d\lambda}{P_{YFP} t_{YFP} \int (X^A_{YFP} M^A_{YFP}) d\lambda}}_{R_P} \right) \tag{5}$$

While R_P may be measured by careful calibration, R_C remains uncertain and dependent on experimental conditions. Note that an additional bias factor from excess free Donor has been neglected this form as it is expected to be minor and is impractical to calibrate ($\frac{C^D}{C^F}$ would be multiplied by the entire right hand expression, C^F being the true concentration of working FRET pairs). In this case, because we have available a reliable inhibitor of MEK, we account for variation in R_C via normalization to a well-inhibited condition at the end of every live cell experiment. Saturating MEK or ERK inhibition yields approximately zero active ERK and zero phosphorylated EKAR (verified by Phos-Tag western blot). In this fully inhibited state, the fraction associated (Ef_A) is expected to be a consistent characteristic of the sensor (k_{au}), the fraction of sensor molecules associated when unphosphorylated (see following section). This value ($k_{au} \cong 0.25$) is estimated by measurements of both inhibitor treated cells, and cells expressing a mutant sensor that cannot be phosphorylated (averaged to limit bias from R_C). We therefore normalize across cells and experiments by

dividing the CFP/YFP ratio for each cell by its value under saturating inhibition and scaling the result against the estimated ideal value for CFP/YFP of the inhibited sensor $(1 - k_{au})$, according to eq. 6.

$$Ef_A = 1 - (1 - k_{au}) \frac{I_{CFP}}{I_{YFP}} \Big/ \left(\frac{I_{CFP}}{I_{YFP}}\right)_{Inh} \tag{6}$$

Inferring ERK activity from EKAR

To interpret ERK activity via the FRET-based reporter, we first consider a model of reporter conformation to estimate the fraction of EKAR that is phosphorylated, and subsequently employ a model of ERK acting on EKAR to estimate ERK activity (relative to competing phosphatases) from the fraction phosphorylated in steady state. A schematic for the combined system is provided in Figure 5A.

Model of FRET reporter conformation states

Above, we related the signal from the EKAR reporter to its association (the conformation bringing the two fluorophores in better alignment for FRET), and that association depends on phosphorylation. Assuming an approximate steady state in reporter phosphorylation, rate equations for association may be written independently for phosphorylated and naïve forms. Here, superscripts A and O represent associated and open conformations, and U and P represent the naïve and phosphorylated forms.

$$\frac{\partial EKAR^{AU}}{\partial t} = k_{au} EKAR^{OU} - k_{ou} EKAR^{AU} \tag{7}$$

$$\frac{\partial EKAR^{AP}}{\partial t} = k_{ap} EKAR^{OP} - k_{op} EKAR^{AP}$$

As both association and phosphorylation are expected to be fast relative to the experiment timeline, the reporter state is assumed to be steady.

$$EKAR^{AU} = \frac{k_{au}}{k_{ou}} EKAR^{OU}, \qquad EKAR^{AP} = \frac{k_{ap}}{k_{op}} EKAR^{OP} \tag{8}$$

The conservation may be applied that total naïve (U) and phosphorylated (P) forms are the sum of the associated and open versions of each, i.e $EKAR^{U} = EKAR^{AU} + EKAR^{OU},\ EKAR^{P} = EKAR^{AP} + EKAR^{OP}$. This allows substitution of the total naïve or phospho- form in lieu of the term for the open conformation.

$$EKAR^{AU} = \frac{k_{au}}{k_{ou}} (EKAR^{U} - EKAR^{AU}) \tag{9}$$

Applying the same for phospho-EKAR, gives new steady state relations. The parameter terms in these relationships may then be lumped for simplicity to give two association parameters that conceptually represent the fractions of EKAR associated when completely unphosphorylated (K_{AU}) and when completely phosphorylated (K_{AP}). These parameters account for the association probability arising from random molecular motion, with and without the phosphorylation present. They are dependent primarily on the structure of the reporter protein itself, though they may be affected by the environment as well.

$$EKAR^{AU} = \frac{k_{au}}{k_{au} + k_{ou}} EKAR^{U}, \qquad EKAR^{AP} = \frac{k_{ap}}{k_{ap} + k_{op}} EKAR^{P} \tag{10}$$

$$K_{AU} = \frac{k_{au}}{k_{au} + k_{ou}}, \qquad K_{AP} = \frac{k_{ap}}{k_{ap} + k_{op}}$$

Combining the equations and using the lumped parameters yields a mixture model for reporter association. Rearrangement gives a linear model for the fraction of reporters in the associated form, in response to the fraction that are phosphorylated. The parameters represent baseline association (without phosphorylation, K_{AU}) and maximal association (when fully phosphorylated, K_{AP}). If the parameters are known, this model may be used to infer the fraction of EKAR that is phosphorylated, based on what fraction is associated.

$$EKAR^{A} = K_{AU}(EKAR^{T} - EKAR^{P}) + K_{AP}EKAR^{P} \tag{11}$$

$$f_A = \frac{EKAR^{A}}{EKAR^{T}} = K_{AU} + (K_{AP} - K_{AU})\frac{EKAR^{P}}{EKAR^{T}}$$

Estimation of EKAR conformation parameters

The lower bound on EKAR association, K_{AU}, was established as previously noted based on saturating MEK inhibition and expression of a mutant EKAR3 reporter with conversion of T422A, and insertion of an alanine after S416 (separating it from the following proline to prevent feasible ERK targeting). Notably, so called "phosphomimetic" mutations (substituting glutamic acid for threonine, e.g.) do not provide the same charge density as a phosphorylation and are not likely to yield the same quantitative effect. The ineffectiveness of glutamic acid substitution in EKAR was verified in MCF-10A cells as the resulting signal was significantly less than that achieved by intact reporters under stimulation.

The upper bound, K_{AP}, was estimated by performing calibration on average population responses via Phos-Tag western blotting for the phosphorylated form of the EKAR sensor. Lysates from the KRAS^{WT}, G12C, G12V, and Q61R cell lines were used, each with baseline (no growth factor) and peak stimulated (10 ng/mL EGF for 15 minutes) conditions (4 replicates per cell line and treatment). After Phos-Tag western blotting, membranes were stained with an anti-GFP antibody (Cell Signaling Technologies #2955) to visualize the EKAR3 reporter, and the average fraction of reporter phosphorylated was quantified. These values were then compared with average fractions associated calculated from the live cell experiments at corresponding treatments and time points. The slope from equation 11 was fit from these data, and $K_{AP} = 0.49$ calculated, allowing a calibrated measurement of the fraction of EKAR3 phosphorylated, based on live-cell measurements.

Model of ERK activity on the FRET reporter

The estimates of fractional phosphorylation of EKAR may be further propagated to estimate the concentration of active ERK in a cell, relative to the competing phosphatase activity. Phospho-EKAR fractions are driven by the amount of active ERK available, as well as the amount of phosphatase competing to dephosphorylate the reporter. The same forces also determine the phospho-state of all other targets of ERK. To finally extend the EKAR measurement to estimate the state of ERK targets in the cell, we use a model of ERK activity and sensor phosphorylation (as a synthetic ERK substrate). The necessary model consists only of the differential equation for the phosphorylation of EKAR, where superscripts indicate the following, P: phosphorylated, U: unphosphorylated, A: active. Parameters are: k_p, the phosphorylation rate by active ERK, and k_{dp}, the dephosphorylation rate due to active competing phosphatases ($PPASE^{A}$, which may vary with

phosphatase expression and activity, etc.). This form assumes rapid kinetics of EKAR association and dissociation, such that conformation does not significantly affect phosphorylation and dephosphorylation rates.

$$\frac{\partial EKAR^P}{\partial t} = k_p ERK^A EKAR^U - k_{dp} PPASE^A EKAR^P \tag{122}$$

Making substitutions to account for total (superscript T) concentrations (which are expected to remain constant on the time scale of reporter measurement) allows for the elimination of unphosphorylated forms from the model.

$$EKAR^T = EKAR^P + EKAR^U \tag{133}$$

$$\frac{\partial EKAR^P}{\partial t} = (EKAR^T - EKAR^P) k_p ERK^A - k_{dp} PPASE^A EKAR^P$$

Since reporter phosphorylation is expected to be fast, relative to the experimental timeline, we solve model for steady state, providing the fraction of EKAR that is phosphorylated, as a function of ERK expression, activity level and phosphatase activity ($k_{dp} PPASE^A$).

$$\frac{EKAR^P}{EKAR^T} = \frac{k_P ERK^A}{k_P ERK^A + k_{dp} PPASE^A} \tag{144}$$

From the EKAR association model, the fraction of reporter molecules associated can be related to fraction phosphorylated, via the two calibration parameters for the minimal and maximal association levels.

$$f_A = \frac{EKAR^A}{EKAR^T} = K_{AU} + (K_{AP} - K_{AU}) \frac{EKAR^P}{EKAR^T} \tag{155}$$

For convenience and clarity, we provide rearrangement steps following the substitution of the EKAR phosphorylated fraction.

$$f_A\left(k_P ERK^A + k_{dp} PPASE^A\right) - K_{AU}\left(\cancel{k_P ERK^A +} k_{dp} PPASE^A\right) = K_{AP} k_P ERK^A - \cancel{K_{AU} k_P ERK^A}$$

$$(f_A - K_{AU}) k_{dp} PPASE^A = (K_{AP} - f_A) k_P ERK^A$$

This leads to the relationship between active ERK levels and the associated fractions of EKAR.

$$\frac{k_P ERK^A}{k_{dp} PPASE^A} = \frac{f_A - K_{AU}}{K_{AP} - f_A} \tag{166}$$

RAS-ERK internal factors model

To compare our observations with previous work in these and similar RAS mutants, we turn to biochemical data previously acquired through purification and in vitro assays. These data allow us to compare our new cell culture and western blot data to the expected results from a cell expressing a single isoform, based on the biochemical activity of that isoform. We prepared a basic model of the internal factors in the RAS-ERK cascade, dependent on the biochemical activities of the RAS isoform. To separate the internal effects on

the core cascade from the external regulatory factors, this model does not include feedback effects or differences between isoforms of RAF, MEK and ERK, but accounts for expression levels and KRAS mutant activities.

$$\frac{\partial Ras^{GTP}}{\partial t} = Ras^{GDP}\left(ke_{int} + ke_{Gef}Gef\right) - Ras^{GTP}(kh_{int} + kh_{GAP}GAP)$$

$$\frac{\partial Raf^{A}}{\partial t} = k_{aRaf}Raf^{I}Ras^{GTP} - Raf^{A}k_{iRaf}$$

$$\frac{\partial MEK^{A}}{\partial t} = k_{aMEK}MEK^{I}Raf^{A} - MEK^{A}k_{iMEK}$$

$$\frac{\partial ERK^{A}}{\partial t} = k_{aERK}ERK^{I}MEK^{A} - ERK^{A}k_{iERK}$$

The total concentrations of each species are substituted to eliminate inactive forms from the model, and the model is solved for the steady state. Total concentrations equal the sum of active and inactive forms, ($X^{Total} = X^{A} + X^{I}$). Psuedo-dissociation constants, ($k_{dX} = k_{iX}/k_{aX}$), are the ratios of the inactivation rate to the activation rate. In the steady state, this model becomes:

$$Ras^{GTP} = Ras^{Total}\frac{ke_{int} + ke_{Gef}Gef}{ke_{int} + ke_{Gef}Gef + kh_{int} + kh_{GAP}GAP}$$

$$Raf^{A} = \frac{Raf^{Total}}{k_{dRaf}/Ras^{GTP} + 1}$$

$$MEK^{A} = \frac{MEK^{Total}}{k_{dMEK}/Raf^{A} + 1}$$

$$ERK^{A} = \frac{ERK^{Total}}{k_{dERK}/MEK^{A} + 1}$$

EKAR signal decay modeling

To estimate phosphatase activity from the decay of EKAR signals, we use a single-exponential model allowing for delayed decay onset and non-zero baseline values.

$$EKAR(t) = \begin{cases} EKAR_0 + a, & t < \tau \\ EKAR_0 + a * e^{-b*(t-\tau)}, & t \geq \tau \end{cases}$$

This model was fit for every single-cell trace, across all cell lines and conditions. Fits were filtered for suitable timing (ensuring that τ was within 45 min of the addition of MEK inhibitor), non-trivial amplitude (a greater than 0.1), and quality (R-squared greater that 0.95).

Western blotting supplemental methods

For pathway expression measurements (Figure 5), samples were mixed 1:1 with a 2x Laemmli sample buffer (100 mM Tris pH 6.8, 4% SDS, 10% Glycerol), heated to 95 C for 5 minutes, cooled and centrifuged briefly. Each well was loaded with 10 uL of prepared sample, and gels were run at 100V until the BPB reached the end of the gel, approximately 90 minutes. Running buffer consisted of 100mM Tris, 292mM Glycine and 0.1% SDS. Transfer was performed in 100mM Tris, 292mM Glycine with 20% MeOH, to nitrocellulose membranes (0.1 um pore). Transfer efficiency was evaluated at several voltages (from 18V to 50V) by Ponceau S and Coomassie Brilliant Blue. Absence of blow-through was verified by Ponceau staining of a second membrane placed behind the first. To maximize transfer efficiency, it was performed at 50V (12.5 V/cm) overnight, with the entire apparatus placed in a refrigerator to control temperature. Transfer buffer was discarded after each use to avoid buildup of contaminants due to the long periods at elevated voltage. Transfer was confirmed by staining with Ponceau S reagent, which is imaged immediately after washing away excess with DI water. To improve quality and lighting uniformity, imaging is performed in a simple photographic light box. Images of Ponceau staining are used to quantify relative protein loading for normalization.

For Phos-Tag separation, sample buffer consisted of 130 mM Tris pH 6.8, 4% SDS (40 mg/mL), 20% glycerol, 200 mM DTT, and ~0.10 mg/mL BPB. Phos-Tag gels were run at 100V until the BPB reached the bottom, approximately 120 minutes. To reliably transfer protein out of the Phos-Tag gels, we first pre-soaked gels in transfer buffer (Tris-Glycine, 20% MeOH) supplemented with 100 mM EDTA twice for 10 minutes each, followed by a 10 minute soak in transfer buffer without EDTA. Transfer is then performed as with SDS-PAGE gels, using the wet tank method, at 50V overnight with the entire apparatus placed inside a refrigerator.

All membranes were blocked in Odyssey® Blocking Buffer (PBS) and probed with the primary antibodies described in Material and Methods and the Reagents and Tools Table. Antigen-antibody complexes were detected using fluorescent goat anti-rabbit or mouse IRDye 800CW and IRDye 680RD secondary antibodies (LI-COR Biosciences) and visualized with a LI-COR Odyssey Classic Infrared Imaging System.

Appendix Table S1: Literature-based values for RAS related biochemical parameters

Parameter	RAS Isoform	Value	Unit	Reference
ke_{int}	KRAS	1.73 ± 0.16 e-3	sec^{-1}	a, b
	G12C	1.00 ± 0.17 e-3		a
	G12D	2.00 ± 0.34 e-3		a
	G12V	8.11 ± 2.47 e-4		a, b
	Q61R	2.93 ± 0.40 e-3		a, b
ke_{Gef}	KRAS	1.03 ± 0.02 e-2	sec^{-1}	b
	G12C	7.51 ± 1.23 e-3		*
	G12D	1.50 ± 0.25 e-2		*
	G12V	9.27 ± 2.64 e-3		b
	Q61R	1.81 ± 0.21 e-2		b
kh_{int}	KRAS	6.8 ± 0.4 e-4	sec^{-1}	a
	G12C	4.9 ± 0.2 e-4		a
	G12D	1.9 ± 0.1 e-4		a
	G12V	4.2 ± 0.2 e-5		a
	Q61R	1.1 ± 0.04 e-5		a
kh_{GAP}	KRAS	4.3 ± 0.1 e-2	sec^{-1}	a
	G12C	2.0 ± 0.3 e-4		a
	G12D	8.9 ± 0.8 e-4		a
	G12V	2.4 ± 0.1 e-4		a

	Q61R	8.5 ± 0.7 e-5		a
k_{dRaf}	KRAS	56 ± 6	nM	a
	G12C	67 ± 12		a
	G12D	270 ± 46		a
	G12V	411 ± 40		a
	Q61R	134 ± 26		a
k_{dMEK}	All	0.36	nM	**
k_{dERK}	All	10	nM	**

a: Hunter et al., 2015; b: Smith et al., 2013; *: Data not directly available – estimated from relative intrinsic rates; **: Parameter fit to match the *fraction* of ERK phosphorylated at Baseline and Steady State for $KRAS^{WT}$ in our Phos-Tag data.

Appendix References

Hoppe, A., Christensen, K. and Swanson, J.A., 2002. Fluorescence resonance energy transfer-based stoichiometry in living cells. *Biophysical journal*, *83*(6), pp.3652-3664.

Hunter, J.C., Manandhar, A., Carrasco, M.A., Gurbani, D., Gondi, S., and Westover, K.D. (2015). Biochemical and structural analysis of common cancer-associated KRAS mutations. Mol Cancer Res 13, 1325-1335.

Smith, M.J., Neel, B.G., and Ikura, M. (2013). NMR-based functional profiling of RASopathies and oncogenic RAS mutations. Proc Natl Acad Sci U S A 110, 4574-4579.
