# Supplementary material for: Oncogenic mutant RAS signaling activity is rescaled by the ERK/MAPK pathway
Source: Mol Syst Biol. 2020 Oct 19;16(10):e9518. doi: 10.15252/msb.20209518 (PMC7569415; doi:10.15252/msb.20209518)
Supplement: Supplementary file 2 — Expanded View Figures PDF [file MSB-16-e9518-s002.pdf]

Expanded View Figures

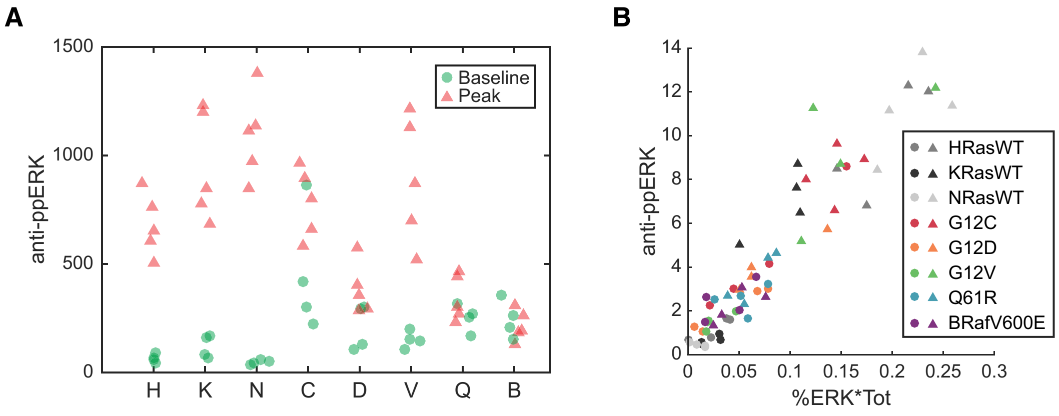

Figure EV1. Correlation of ppERK measured by anti-ppERK antibody and calculated from Phos-Tag.

A ppERK measured in baseline and peak stimulated samples of each cell line, via immuno blot with anti-ppERK antibody.

B Data from A, plotted against the ppERK calculated for the same samples, as percent of ERK phosphorylated (via Phos-Tag) times the total ERK (via immuno blot with anti-ERK1/2 antibody).

 © 2020 The Authors
