# Supplementary figures and images for: Oncogenic mutant RAS signaling activity is rescaled by the ERK/MAPK pathway
Source: Mol Syst Biol. 2020 Oct 19;16(10):e9518. doi: 10.15252/msb.20209518 (PMC7569415; doi:10.15252/msb.20209518)

Western Blot Source Data for Figure 4

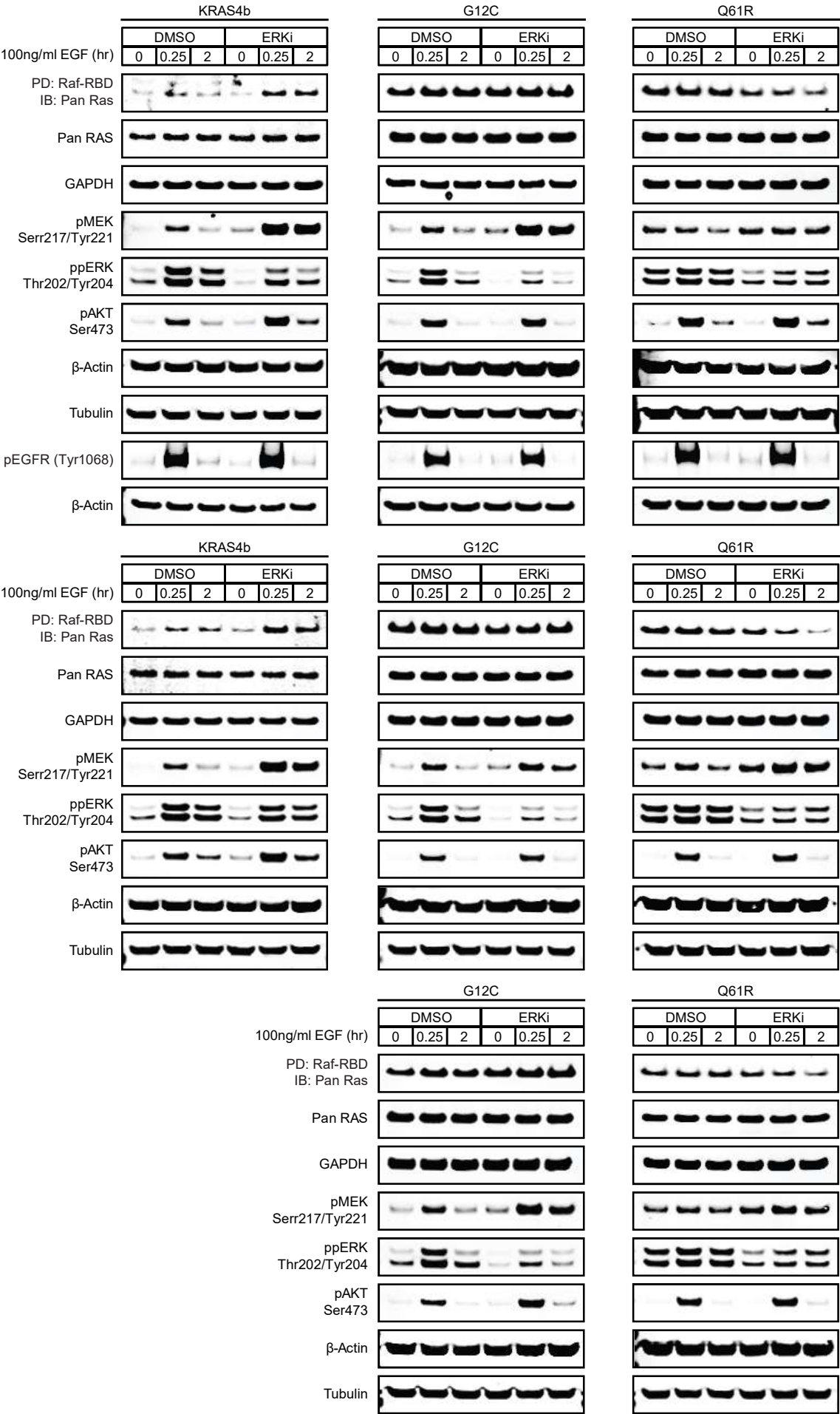

Supplement: Supplementary file 4 — Source Data for Figure 4 [file MSB-16-e9518-s004.zip › Gillies2020_SourceData_Fig4.pdf]
