# Supplementary material for: Oncogenic mutant RAS signaling activity is rescaled by the ERK/MAPK pathway
Source: Mol Syst Biol. 2020 Oct 19;16(10):e9518. doi: 10.15252/msb.20209518 (PMC7569415; doi:10.15252/msb.20209518)

Ras, MEK, ERK expression after 0, 15m EGF (10 ng/mL)
Replicates 1-3

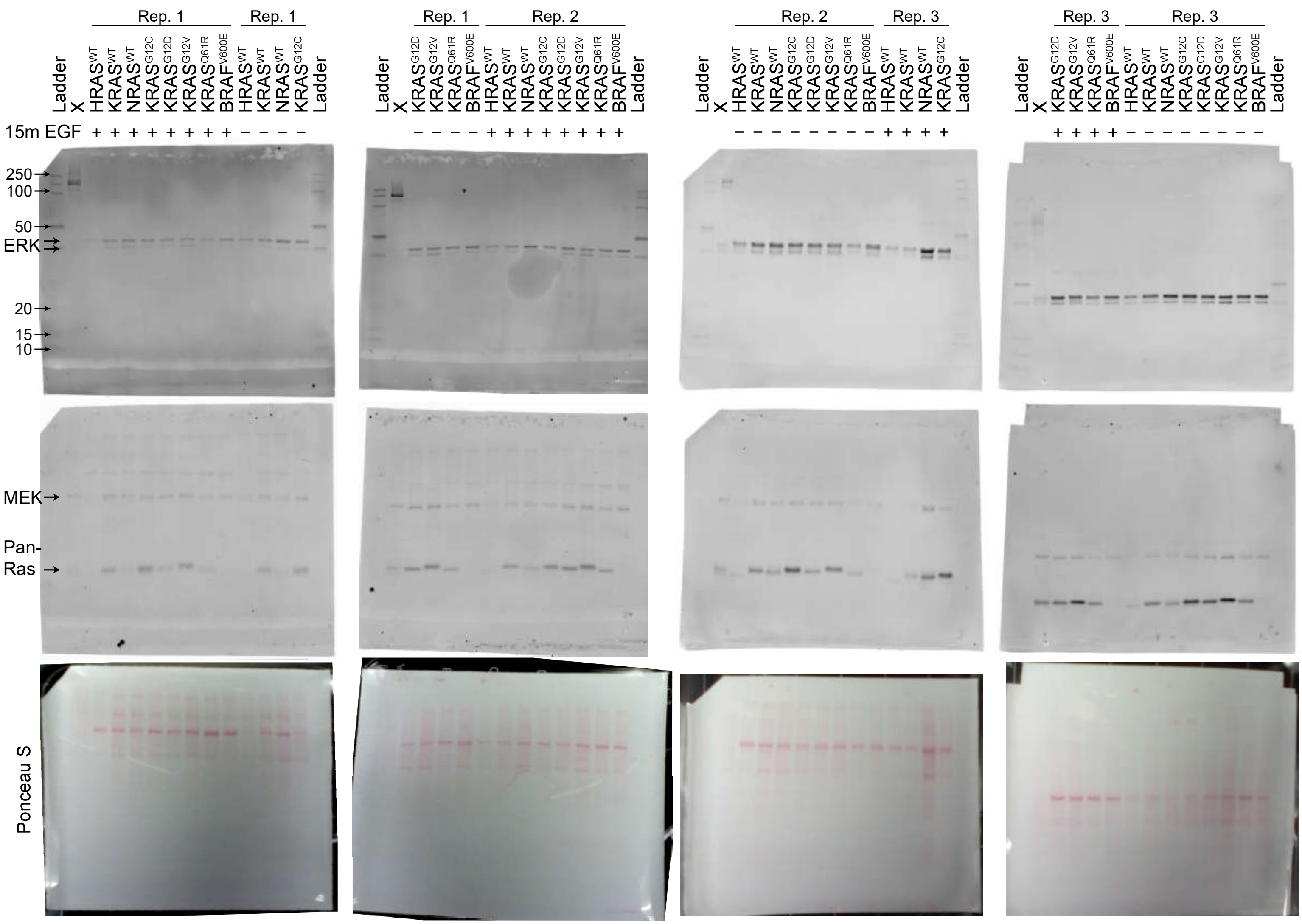

BRaf, CRaf expression after 0, 15m EGF (10 ng/mL)
Replicates 1-3

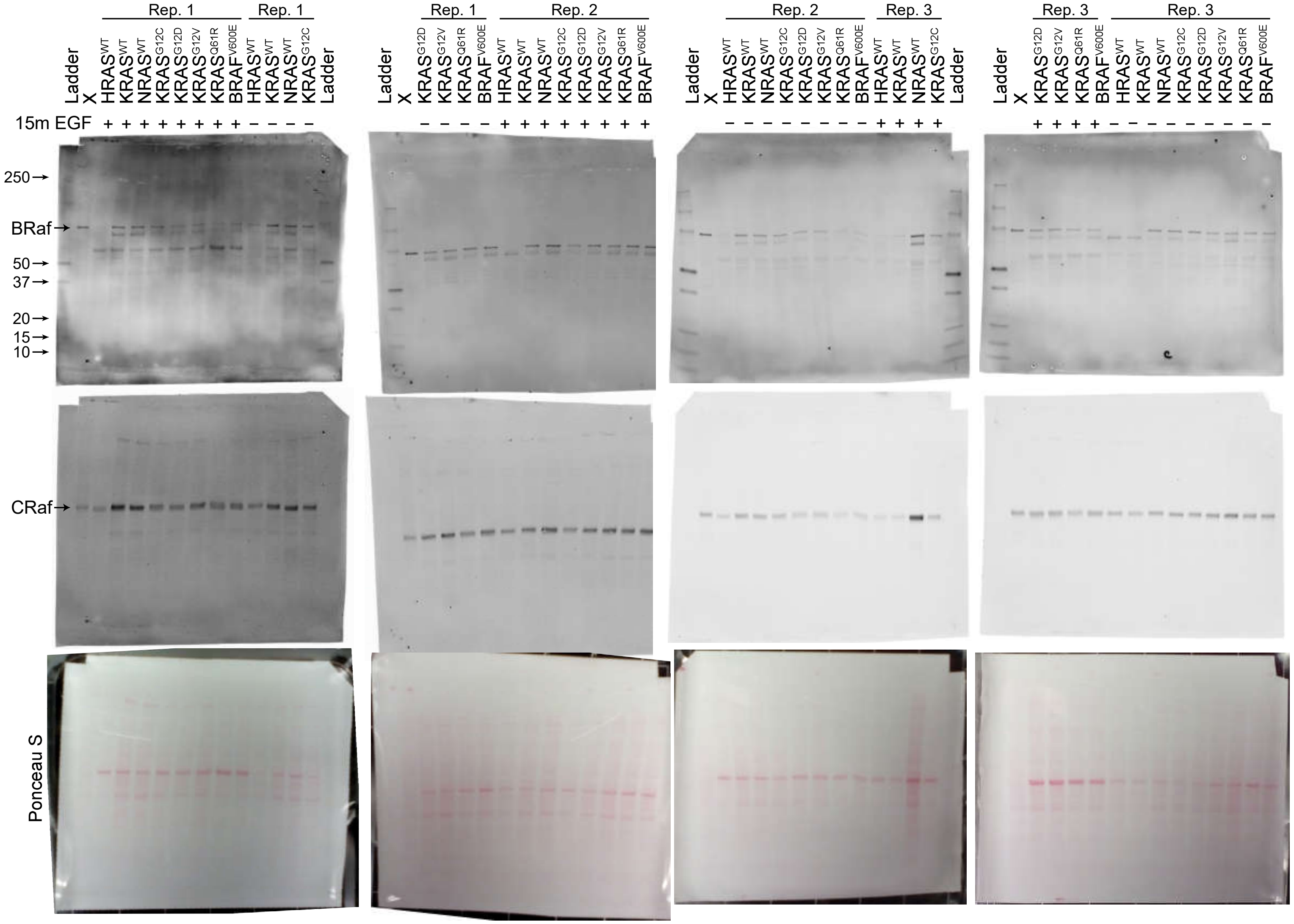

Ras, BRaf, CRaf, MEK, ERK expression after 0, 15m EGF (10 ng/mL)
Replicate 4, and misc. repeats

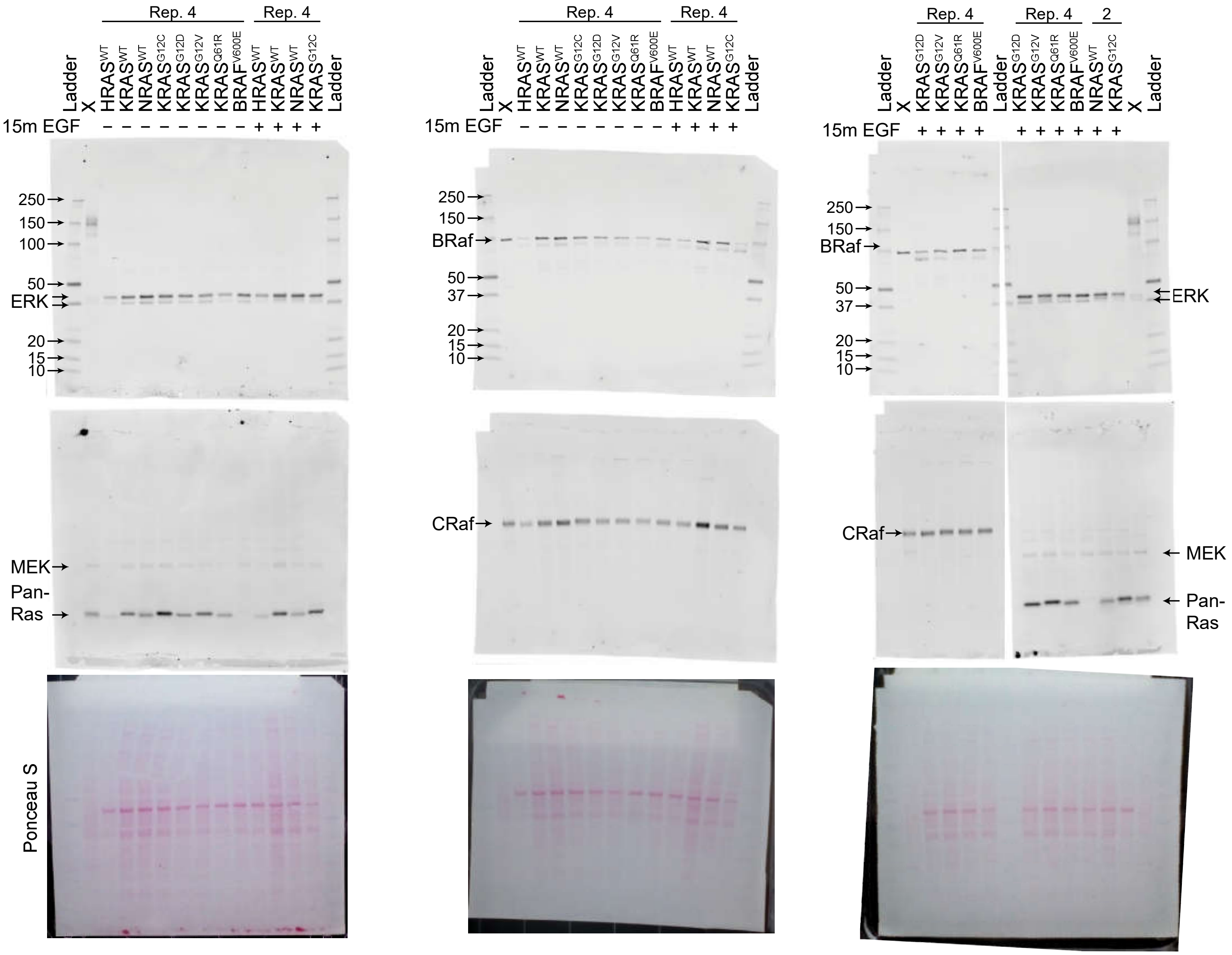

ERK(total) and ppERK expression after 0, 15m EGF (10 ng/mL)
Replicates 1-3, $KRAS^{G12V}$ Rep. 2, 0 EGF used for cross-blot normalization

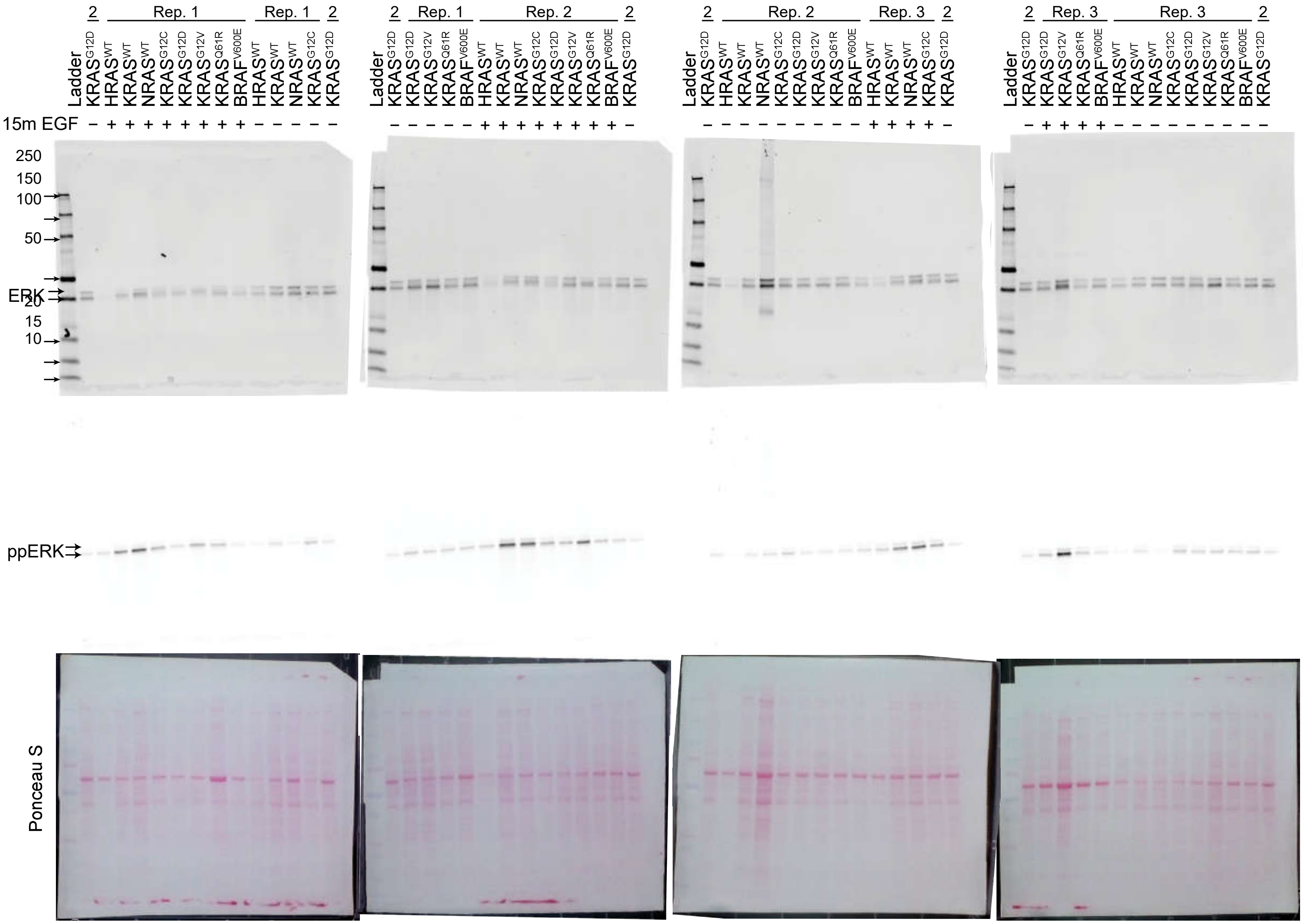

ERK(total) and ppERK expression after 0, 15m EGF (10 ng/mL)
Replicates 3-4, KRAS^{G12V} Rep. 2, 0 EGF used for cross-blot normalization

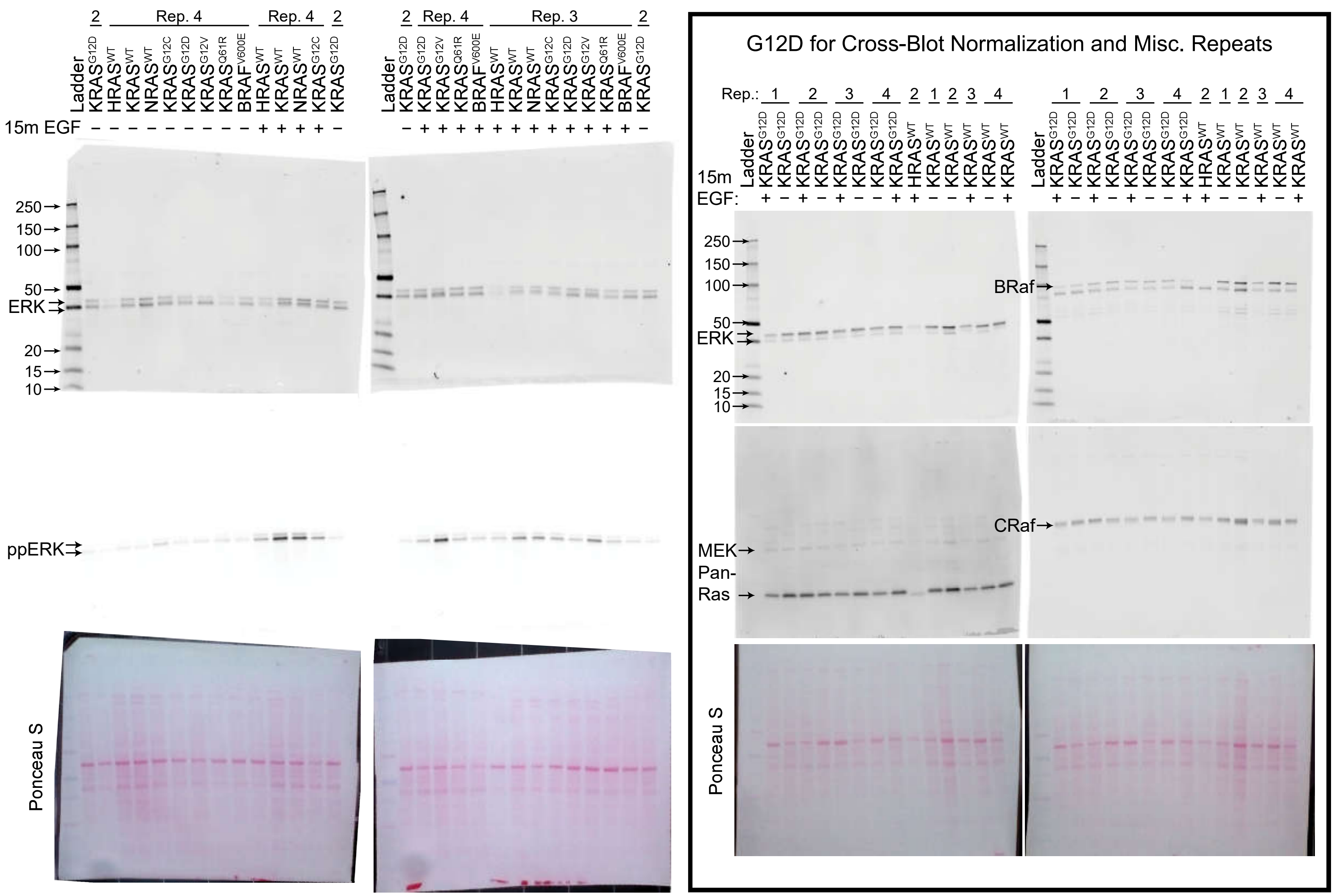

Steady state Ras, Raf, MEK, ERK expression, after 2h 10 ng/mL EGF (+).
Also includes: (-) 0 EGF, (*) 15 min EGF, or (i) 2 hr 100 nM MEKi (PD325901)

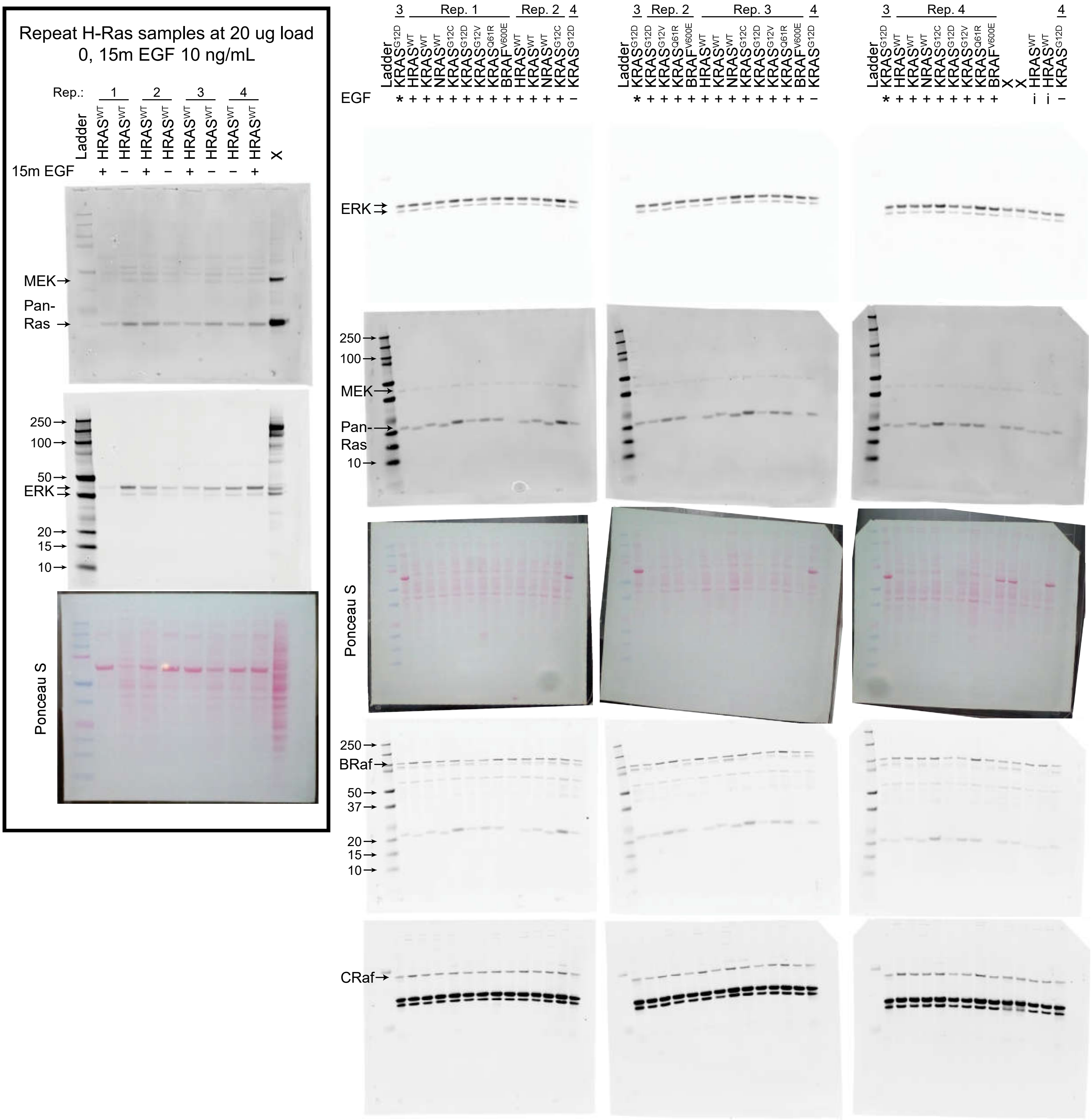

Phos-Tag Blot for ERK
Includes: (-) 0 EGF, (+) 15 min or 2 hr 10ng/mL EGF, or (i) 2 hr 100 nM MEKi (PD325901)

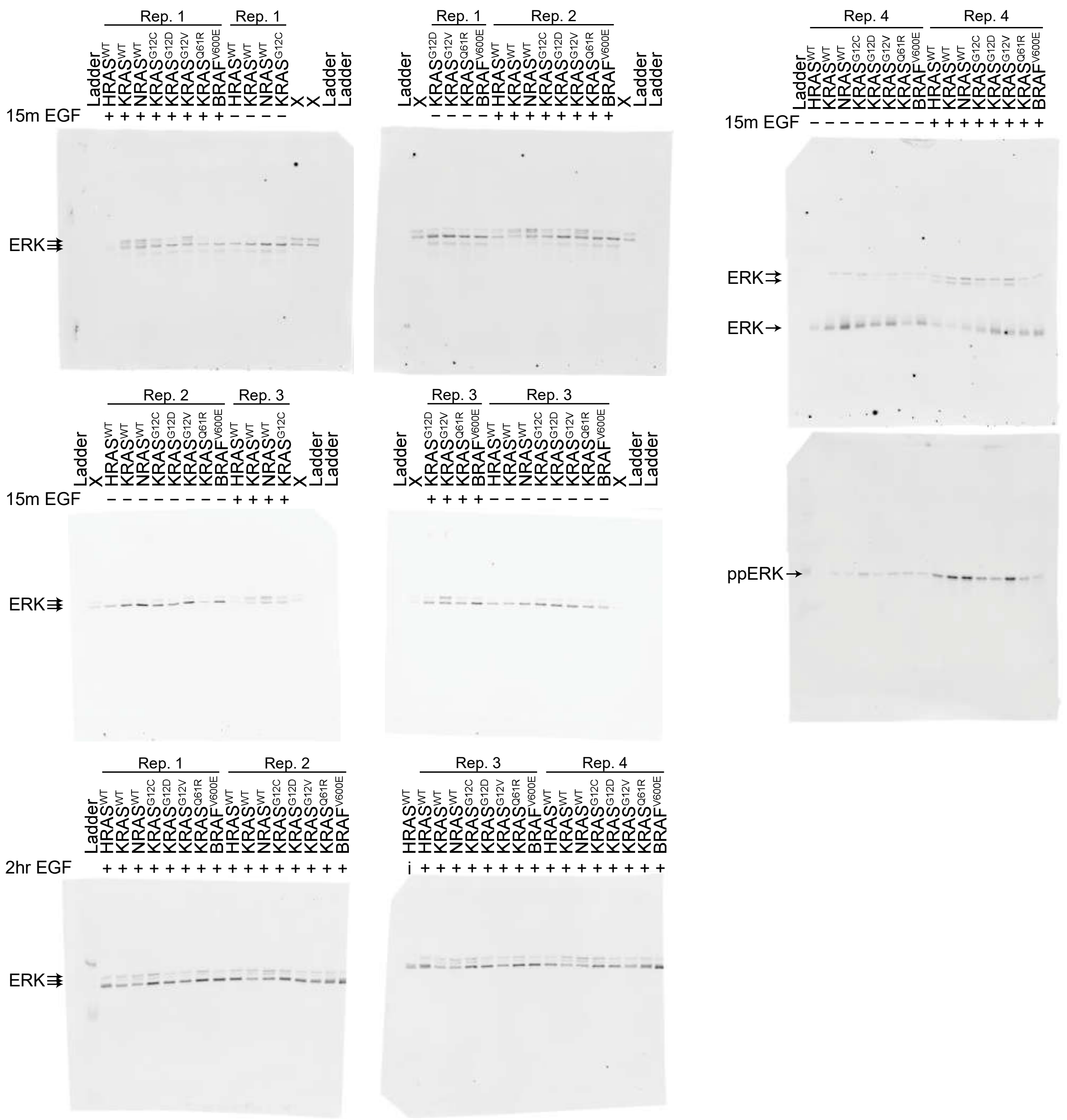

Phos-Tag Blot of EKAR3 for reporter calibration
IB: αGFP

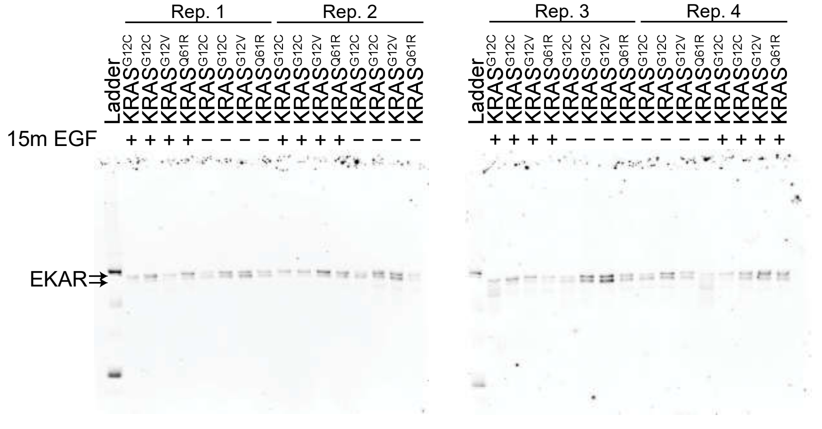

Supplement: Supplementary file 5 — Source Data for Figure 5 [file MSB-16-e9518-s005.zip › Gillies2020_Fig5_SourceData.pdf]
